# Nanotechnology-Based Delivery Systems and Retinal Pigment Epithelium: Advances, Targeting Approaches, and Translational Challenges

**DOI:** 10.3390/biom15111592

**Published:** 2025-11-13

**Authors:** Michele Nardella, Marco Pellegrini, Angeli Christy Yu, Ginevra Giovanna Adamo, Marco Mura, Massimo Busin

**Affiliations:** 1 Department of Translational Medicine, University of Ferrara, 44121 Ferrara, Italyangelichristy.yu@unife.it (A.C.Y.);; 2 Department of Ophthalmology, Ospedali Privati Forlì “Villa Igea”, 47122 Forlì, Italy; 3 Ophthalmology Clinic Operating Unit, Head and Neck Department, Sant’Anna University Hospital, 44124 Ferrara, Italy; 4 Istituto Internazionale per la Ricerca e Formazione in Oftalmologia (IRFO), 47122 Forlì, Italy; 5 Department of Vitreoretinal and Uveitis Divisions, King Khaled Eye Specialist Hospital, Riyadh 11462, Saudi Arabia

**Keywords:** retinal pigment epithelium, age-related macular degeneration, nanotechnology, drug delivery, gene therapy

## Abstract

The retinal pigment epithelium (RPE) is essential for maintaining retinal integrity, and its dysfunction underlies several progressive ocular diseases, including age-related macular degeneration, choroidal neovascularization (CNV), inherited retinal disorders (IRDs), and proliferative vitreoretinopathy (PVR). Although current therapies have improved disease management, they mainly target secondary pathological mechanisms and do not directly preserve or restore RPE function. Moreover, the delivery of therapeutic molecules or genes to the RPE remains a major challenge due to the presence of multiple ocular barriers and the need for sustained, localized action. Nanomedicine offers innovative solutions to these limitations by enabling precise, controlled, and cell-specific delivery of drugs and genetic materials. Engineered nanocarriers can be optimized to traverse ocular barriers, enhance bioavailability, and modulate the retinal microenvironment. This review summarizes recent advances in nanoscale delivery systems for RPE-targeted therapies, focusing on design principles, targeting strategies, and therapeutic applications, and discusses the translational challenges that must be addressed to bring nanotechnology-based treatments closer to clinical application.

## 1. Introduction

The Retinal Pigment Epithelium (RPE) is a monolayer of pigmented cells located between the neural retina and the choroid, playing a pivotal role in retinal homeostasis. It is responsible for numerous physiological processes, including nutrient and ion transport, phagocytosis of photoreceptor outer segments, light absorption, maintenance of the visual cycle, protection against photo-oxidative damage, secretion of growth factors, and preservation of the external blood–retinal barrier (BRB) [1]. Given its essential role, RPE dysfunction is a central feature in the pathogenesis of several vision-threatening diseases, such as age-related macular degeneration (AMD), choroidal neovascularization (CNV), inherited retinal disorders (IRDs), proliferative vitreoretinopathy (PVR) [2].

Despite significant progress in understanding the molecular mechanisms underlying these disorders, current therapeutic options remain limited. Most available treatments provide only symptomatic relief or require frequent administration, failing to address the underlying cellular degeneration. For instance, intravitreal injections of anti-VEGF agents—currently the standard of care for neovascular AMD—effectively control neovascularization but impose a substantial burden on patients and healthcare systems due to the need for lifelong, repetitive dosing [3]. In parallel, major advances have been achieved in gene therapy for IRDs, where the RPE has emerged as a validated therapeutic target. The approval of voretigene neparvovec (Luxturna) for RPE65-associated Leber congenital amaurosis demonstrated that direct gene supplementation can restore visual function in humans [4]. However, viral vectors suffer from limitations including restricted packaging capacity, potential immunogenicity, and high manufacturing costs, underscoring the need for complementary delivery strategies that are safer, redosable, and capable of carrying complex genetic payloads [5].

A major challenge across all RPE-related conditions lies in the intrinsic complexity of ocular delivery. The RPE is protected by multiple anatomical and physiological barriers—including the vitreous gel, internal limiting membrane (ILM), Bruch’s membrane (BM), and the tight junctions of the BRB—that collectively restrict drug penetration, stability, and persistence at the target site [6]. These barriers often lead to subtherapeutic concentrations, accelerated clearance, or off-target distribution, limiting the efficacy of conventional formulations [7].

To overcome these limitations, nanotechnology has gained traction as a promising approach for developing targeted and sustained ocular therapies. Recent advances in nanoparticle engineering have demonstrated the ability to enhance ocular bioavailability, extend drug residence time, and enable selective delivery to RPE cells through physicochemical or ligand-mediated mechanisms [8]. Recent studies have further validated the therapeutic potential of nanocarrier-based systems for retinal and RPE targeting, demonstrating prolonged release, improved biodistribution, and enhanced safety profiles in both preclinical and translational models [9,10,11].

This review provides an updated and critical overview of nanotechnology-based delivery systems targeting the RPE, emphasizing their potential to overcome ocular barriers, improve therapeutic precision, and enable clinical translation. By adopting a broad and integrative perspective, the work examines how advances in nanomedicine can be applied to a range of RPE-related conditions and therapeutic modalities, spanning both drug and gene delivery approaches. Through this comprehensive analysis, the review outlines emerging concepts and challenges that are shaping the future of RPE-targeted nanomedicine.

## 2. Materials and Methods

A comprehensive literature search was conducted to identify peer-reviewed studies investigating nanotechnology-based therapeutic strategies for RPE-related diseases. The search was performed across major scientific databases, including PubMed, Scopus, and Google Scholar. To ensure broad coverage, both controlled vocabulary terms and free-text keywords were used. For studies on drug delivery, the search combined terms such as “nanoparticles” or “nanocarriers” with “retinal pigment epithelium” and “drug delivery.” For gene therapy approaches, the search incorporated keywords including “gene therapy”, “nanotechnology”, and “retinal pigment epithelium”, to identify publications addressing the integration of genetic interventions within nanomedical platforms.

The search was limited to English-language articles published up to 31 August 2025, to capture recent and clinically relevant advances. Inclusion criteria prioritized studies examining molecular mechanisms, therapeutic strategies, or preclinical and clinical outcomes specifically related to RPE dysfunction and regeneration. Eligible publications encompassed in vitro and in vivo experimental studies, clinical trials, systematic reviews, meta-analyses, and cohort studies. Particular emphasis was placed on high-quality research investigating innovative drug- and gene-delivery systems targeting the RPE and its associated pathogenic pathways.

## 3. Therapeutics Delivery to the RPE

**Figure 1:**
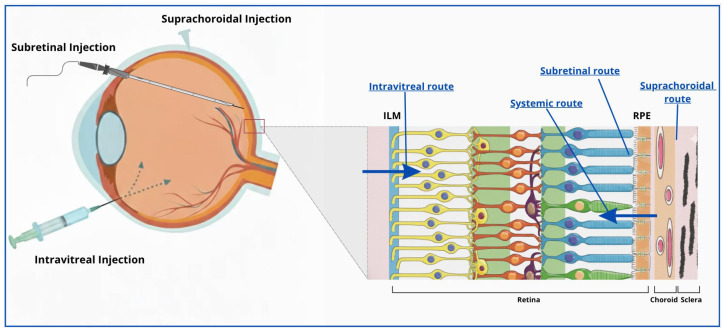
Schematic illustration of the main RPE-targeted delivery routes—intravitreal, subretinal, and suprachoroidal—showing their anatomical access to the posterior segment.

The delivery of therapeutic agents to the RPE represents a considerable challenge in ophthalmology. The eye is a small yet highly compartmentalized organ, with multiple anatomical and physiological barriers that preserve visual function but simultaneously restrict drug access, bioavailability, and residence time at the target site. These same barriers that maintain ocular homeostasis complicate the pharmacological treatment of RPE-related diseases demanding administration routes capable of achieving therapeutic concentrations in the posterior segment without compromising ocular safety [7]. Table 1. provides a comparative overview of the principal ocular drug delivery routes, whereas Figure 1. shows their corresponding anatomical access to the posterior segment. 

Topical administration, typically in the form of eye drops, remains the standard approach for anterior segment diseases owing to its simplicity, noninvasiveness, and high patient compliance. However, its utility for RPE-targeted therapy is extremely limited. Following instillation, most of the applied dose is lost through tear turnover, blinking, and nasolacrimal drainage, with less than 5% reaching intraocular tissues. Multiple static and dynamic barriers—such as the corneal epithelium, conjunctiva, and aqueous humor—impede posterior diffusion, making topical delivery unsuitable for achieving therapeutic concentrations at the RPE [12].

Periocular administration, including subconjunctival and sub-Tenon’s injections, has been explored to bypass the corneal epithelium and deposit the drug closer to the sclera [13]. Although moderately invasive, these routes offer only partial improvement in posterior-segment delivery. The low permeability of the scleral extracellular matrix and the rapid clearance of drugs via episcleral and choroidal circulation substantially limit bioavailability [14]. More recently, suprachoroidal injection, enabled by microneedle-assisted delivery, has gained attention as a minimally invasive technique that can deposit therapeutic agents near the RPE–choroid complex [15]. The suprachoroidal space allows circumferential diffusion around the posterior pole with reduced anterior exposure, providing a compromise between safety and efficacy. However, limitations remain, including non-uniform distribution, potential drug clearance through the choriocapillaris, and the need for dedicated injection devices. Despite these constraints, its minimally invasive nature and expanding preclinical evidence make the suprachoroidal route a promising option for sustained posterior delivery [16].

Intravitreal injection currently represents the gold standard for posterior-segment therapy [17]. By directly introducing drugs into the vitreous cavity, it bypasses anterior barriers and achieves therapeutic concentrations near the retina and RPE. However, the vitreous matrix—composed of collagen and hyaluronic acid (HA)—slows the diffusion of large or charged molecules, while the ILM further restricts penetration into deeper retinal layers [18]. Small molecules are rapidly cleared, and biologics such as anti-VEGF antibodies have longer half-lives yet still require repeated dosing every four to eight weeks to maintain efficacy [19]. The procedure’s simplicity, repeatability, and outpatient feasibility make it the preferred route for chronic treatment. Its overall complication rate is low: infectious events such as endophthalmitis are exceedingly rare with proper asepsis, while non-infectious complications like retinal detachment occur only sporadically and are typically related to procedural factors rather than the injection itself [20,21].

Subretinal injection provides the most direct access to the RPE by delivering the therapeutic agent into the potential space between the photoreceptor outer segments and the RPE monolayer [22]. It represents the most effective approach for gene therapy targeting the RPE, as demonstrated by the clinical success of voretigene neparvovec in RPE65-associated Leber congenital amaurosis [4]. By placing the vector in direct contact with RPE and photoreceptors, subretinal delivery achieves high transduction efficiency while the confined, relatively immune-privileged environment favors stable expression and minimizes inflammatory responses. In contrast, intravitreal injection is limited by diffusion barriers and vitreous exposure, which can dilute the vector and increase immune activation [5]. Despite its advantages, subretinal administration remains surgically invasive, requiring pars plana vitrectomy, localized retinal detachment, and precise microcannula injection, all demanding an experienced vitreoretinal surgeon [23]. Intraoperative risks include subretinal hemorrhage, macular hole formation, and iatrogenic retinal tears, while postoperative complications may involve transient inflammation or retinal thinning. Although infection risk after vitrectomy is low [24], the need for specialized facilities and personnel limits its applicability for chronic or large-field treatments. Nevertheless, unlike intravitreal therapy, it is typically performed as a single procedure with durable, long-lasting effects [25].

Finally, systemic administration, either oral or intravenous, offers a noninvasive route and allows repeated dosing, but its efficiency in reaching the posterior segment is poor. Although the choriocapillaris is highly vascularized, its fenestrations are covered by diaphragms that restrict the passage of large or hydrophilic molecules [26,27]. Consequently, achieving therapeutic levels at the RPE often requires high systemic doses that increase the risk of toxicity and off-target effects. Moreover, age- and disease-related changes—such as BM thickening or lipid accumulation—can further reduce permeability and create interindividual variability [28,29].

## 4. Nanomedicine Approaches for RPE-Related Diseases

Nanomedicine represents a rapidly evolving field at the intersection of materials science, pharmacology, and molecular biology, aiming to precisely modulate biological processes at the cellular and subcellular levels [30]. The application of nanotechnology to ocular pharmacotherapy exploits nanoscale carriers that improve stability against enzymatic degradation, enable controlled or stimuli-responsive release, and enhance tissue-specific delivery [31]. By traversing ocular diffusion barriers and prolonging residence time at the target site, nanoparticles can achieve sustained and effective therapeutic exposure while reducing the need for repeated dosing [32]. These features are particularly valuable for chronic degenerative and neovascular diseases involving the RPE, where long-term therapeutic modulation is required to preserve vision [33].

Preclinical studies have demonstrated that nanotechnology-based delivery systems can overcome several limitations of conventional ocular pharmacotherapy [34,35]. Most investigations on RPE-targeted nanomedicine have employed intravitreal administration, reflecting the current clinical standard for delivering biologics to the posterior segment. Experimental evidence shows that intravitreally injected nanoparticles can successfully transport small molecules, peptides, proteins, and nucleic acids to retinal tissues [17,36,37]. Notably, although microparticle formulations have entered clinical testing for diabetic retinopathy, no trials have yet evaluated intravitreally delivered nanoparticles for RPE-related diseases, underscoring the translational gap between preclinical innovation and clinical application [17].

Other administration routes have also been explored [34]. Subretinal delivery provides direct access to the RPE and is particularly suitable for gene and nucleic acid therapies due to its high transfection efficiency [38]. Topical and systemic formulations are likewise under investigation; while their ability to reach the posterior segment remains limited, surface-engineered nanocarriers (e.g., HA-modified systems) have shown improved penetration profiles [39,40]. Moreover, a small number of early Phase I clinical trials are currently assessing topical nanocarrier-based formulations for AMD, reflecting a growing interest in non-invasive approaches for posterior segment therapy [19].

Despite these advancements, achieving efficient and selective delivery to the RPE remains a central challenge. Carrier size, surface charge, and functional modifications are being optimized to refine intraocular distribution and reduce off-target interactions [34,35].

Critically, this optimization is influenced by the pathological microenvironment, which alters nanoparticle stability, diffusion, and uptake at disease sites [7]. In diseases such as AMD, CNV, or PVR, the RPE and surrounding tissues undergo profound biochemical and structural alterations that modulate nanoparticle stability, diffusion, and uptake [41]. Oxidative stress is one of the main hallmarks of RPE dysfunction. Excessive production of reactive oxygen species (ROS) leads to lipid peroxidation, protein oxidation, and melanin degradation, which can destabilize lipid-based carriers or alter nanoparticle surface charge [35]. At the same time, this oxidative imbalance can be exploited: redox-responsive nanocarriers can use ROS as a trigger for targeted drug release in affected tissues [42].

Chronic inflammation also plays a crucial role. Activated macrophages and microglia increase nanoparticle clearance, while inflammatory mediators such as TNF-α, IL-1β, and complement proteins disrupt the outer BRB, changing diffusion and immune responses [43].

Structural changes in the extracellular matrix further influence drug delivery. Thickening and lipid accumulation in BM decrease permeability and receptor accessibility [44], while the formation of new vessels and hypoxia create heterogeneous perfusion and pH variations [45]. These pathological cues can, however, be leveraged for smart drug release through pH- or enzyme-sensitive nanocarriers designed to act preferentially in diseased areas [46].

The following sections will discuss the main classes of nanotechnology systems, strategies to enhance their targeting specificity, and the therapeutic payloads they are designed to deliver.

## 5. Classes of Nanotechnology Systems Reaching the RPE

**Figure 2:**
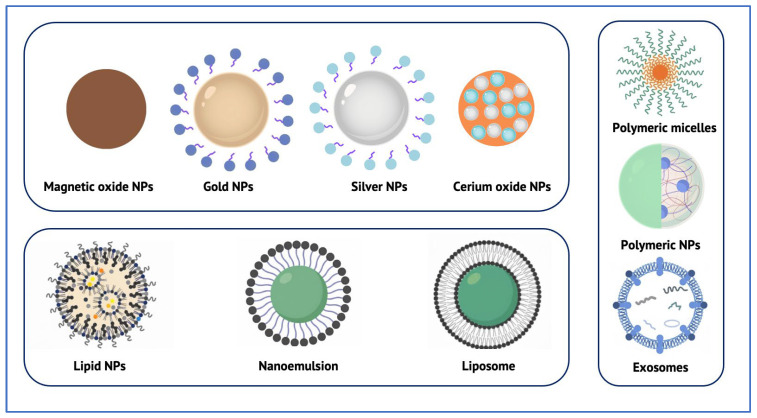
Schematic representation of the main classes of nanocarriers investigated for RPE–targeted therapy. Abbreviation: NPs—Nanoparticles.

Nanoparticles constitute the foundation of nanotechnology-based strategies for targeting the RPE [28]. The main classes of nanocarriers investigated for RPE-targeted therapy are illustrated in Figure 2., while their representative characteristics are summarized in Table 2. Typically ranging from 10 to 500 nm, these carriers can encapsulate, adsorb, or conjugate a wide variety of therapeutic agents—including small molecules, proteins, nucleic acids, and gene-editing tools—while protecting them from enzymatic degradation and controlling their release [36]. Recent research has increasingly focused on nanocarriers designed to overcome the structural and biological barriers that separate the RPE from accessible ocular compartments, with particular emphasis on sustained-release and biocompatible formulations validated in preclinical models [36,47].

Among these, polymeric nanoparticles are the most extensively studied [48,49,50]. Biodegradable systems based on poly(lactic-co-glycolic acid) (PLGA) or polyethylene glycol (PEG) demonstrate prolonged intraocular residence and efficient RPE uptake after intravitreal or subretinal injection [50]. For example, PEG–PLGA nanoparticles loaded with dexamethasone maintained therapeutic efficacy for up to eight weeks in rodent models of retinal degeneration, significantly reducing injection frequency compared with free drug delivery [48]. Chitosan- and HA-modified nanoparticles further enhance cellular adhesion and muco-penetration, promoting interaction with the apical RPE surface. These findings highlight the potential of polymeric systems to sustain therapeutic exposure and address one of the principal limitations of current anti-VEGF and corticosteroid therapies—the need for repeated intravitreal injections [49,50]. Concerns remain regarding unintended effects in surrounding tissues or systemic exposure. Unlike larger PLGA constructs used in other biomedical applications, the safety and biocompatibility of very small nanoparticles still require further evidence [51].

Lipid-based nanoparticles (LNPs), including solid lipid nanoparticles (SLNs) and nanostructured lipid carriers (NLCs), have gained attention due to their biocompatibility and ability to encapsulate both hydrophilic and hydrophobic molecules [52,53]. In RPE-targeted studies, SLNs showed prolonged release and improved bioavailability following intravitreal injection, while NLCs exhibited higher loading capacity and enhanced physical stability [54]. Lipid nanoparticles are also emerging as non-viral vectors for ocular gene delivery, particularly via the subretinal route, enabling mRNA or plasmid transfection in RPE cells with minimal immunogenicity compared to AAV vectors [55]. However, challenges persist in terms of large-scale reproducibility and storage stability [56]. However, manufacturing reproducibility and storage stability remain key translational challenges [57].

Metallic and inorganic nanoparticles, such as gold, cerium oxide, and mesoporous silica, have also been evaluated for their antioxidant and anti-inflammatory potential in RPE degeneration [58]. Gold nanoparticles functionalized with anti-VEGF peptides demonstrated localized inhibition of choroidal neovascularization after a single intravitreal injection, while cerium oxide nanoparticles exhibited intrinsic ROS scavenging activity, prolonging photoreceptor survival in AMD models [58,59]. These systems may permit infrequent dosing but raise concerns regarding long-term accumulation, as metallic cores are non-biodegradable [60]. Mesoporous silica nanoparticles, by contrast, offer tunable porosity and high loading capacity, although surface modification is often required to mitigate cytotoxicity [61].

Liposomes and polymeric micelles remain versatile soft-matter carriers for ocular drug delivery. Liposomes, owing to their phospholipid bilayers, can co-encapsulate hydrophilic and hydrophobic drugs and are naturally taken up by RPE cells via phagocytosis [62]. Their components are metabolized after disintegration, resulting in generally low toxicity. Various intravitreally administered liposomal formulations have enabled sustained release of anti-VEGF agents for over one month in animal models. However, their stability in vivo is easily influenced by enzymatic activity, pH changes, and phagocytosis by macrophages, leading to rapid clearance and the need for repeated dosing [63]. Polymeric micelles offer greater structural stability and customizable surface chemistry for ligand-mediated targeting, but premature drug leakage can occur if not properly cross-linked [64].

Extracellular vesicles—particularly exosomes derived from retinal or mesenchymal cells—represent a promising new generation nanocarrier strategy. Their endogenous origin confers biocompatibility, low immunogenicity, and intrinsic tropism toward retinal tissues [65]. Engineered exosomes carrying siRNA or antioxidant peptides have achieved efficient RPE delivery after intravitreal administration without inducing inflammation. However, large-scale production, purification, and stability remain significant barriers to translation [66].

From a clinical standpoint, the advantages of RPE-targeted nanocarriers include reduced injection frequency through sustained release, improved target selectivity, and potential minimization of surgical invasiveness compared with repeated subretinal procedures [38,67]. Conversely, limitations include the need for optimized biocompatibility, the possibility of chronic accumulation for non-degradable systems, and uncertainties regarding long-term safety in human eyes. While polymeric and lipid nanoparticles are advancing toward clinical translation, metallic and exosome-based systems still require comprehensive toxicological assessment and scalable manufacturing before human application [30,67].

**Table 2:** Nanocarrier classes for RPE-targeted delivery, with typical therapeutic cargo, common routes of ocular administration, key advantages and limitations.

Nanocarrier Class	Typical Therapeutic Cargo	Common Routes of Ocular Administration	Advantages	Limitations/Considerations
Polymeric nanoparticles (e.g., PLGA, PEG–PLGA, chitosan-, HA-modified)	Anti-VEGF drugs, siRNA, miRNA, proteins [48]	Intravitreal; Subretinal [49,50] Systemic [68]	Sustained release; Reduced injection frequency; Tunable surface chemistry; Good cellular internalization [49,50]	Size-dependent drug loading; Risk of burst release; Limited long-term safety data for very small particles; Potential for off-target accumulation [51]
Lipid-based nanoparticles (SLNs, NLCs)	Hydrophobic and hydrophilic drugs; mRNA; Plasmids [52,53]	Intravitreal [53]; Subretinal [55]; Topical [69]	High biocompatibility; Suitable for gene delivery; Good loading efficiency [52,53]	Challenges in large-scale reproducible manufacturing; Storage instability; Risk of rapid clearance [56]
Metallic and inorganic nanoparticles (e.g., Gold, Cerium oxide, Mesoporous silica)	Anti-VEGF drugs; Antioxidants; Enzyme mimetics [58]	Intravitreal [58]; Systemic [70]; Topical [71]	Potential dose sparing; Intrinsic bioactivity (e.g., ROS scavenging); Long-lasting effects [60]	Non-biodegradable core may accumulate; Limited data on long-term ocular safety; Risk of lysosomal persistence [61]
Liposomes	Anti-VEGF drugs; Steroids; Proteins [62]	Intravitreal [62]; Subretinal [63]; Systemic [68]	Co-encapsulation of hydrophilic & hydrophobic agents; Generally low toxicity; Potential for sustained release [62]	Sensitive to pH and enzymatic degradation; Rapid vitreous clearance; May still require repeated injections [63]
Polymeric micelles	Hydrophobic small molecules; peptides [64]	Intravitreal [64]	High solubilization capacity; Customizable targeting ligands; Good stability in solution [64]	Possible premature drug leakage; Requires cross-linking for durable release [64]
Extracellular vesicles/Exosomes	siRNA; miRNA; proteins; antioxidants [65]	Intravitreal [65]	Endogenous biocompatibility; Low immunogenicity; Natural tropism to retinal cells [65]	Scalability and purification challenges; Cargo loading variability; Stability and dosing protocols not yet standardized [66]

## 6. Targeting Strategies for the RPE

Efficient and selective drug delivery to the RPE requires nanocarriers not only to overcome ocular barriers but also to incorporate design features that enhance their accumulation and cellular uptake within this specialized monolayer [18]. Nanotechnology-based approaches often exploit the intrinsic phagocytic activity of the RPE cells, which naturally internalizes both endogenous substrates, such as photoreceptor outer segments, and exogenous nanoscale particles [72,73]. To achieve controlled therapeutic action in terms of location, timing, and dosage, designing nanocarriers that respond to the pathological microenvironment of retinal degenerative diseases has become an increasingly appealing strategy [35,59]; The following subsections discuss key targeting strategies, including physicochemical tuning, ligand–receptor-mediated approaches, and pathology-responsive drug release mechanisms.

### 6.1. Physicochemical Tuning

The physicochemical characteristics of nanoparticles—particularly size, surface charge, and mechanical properties—are key determinants of their intraocular distribution, clearance, and cellular uptake following intravitreal or systemic administration [18].

In intravitreal delivery, particle size critically influences diffusion through the vitreous and penetration into retinal tissues. Large nanoparticles (>1000 nm) exhibit severely restricted diffusion and often remain entrapped within the vitreous for extended periods. Although this depot-like behavior may support sustained-release formulations, it limits access to deeper layers such as the RPE. By contrast, smaller particles (<1000 nm) display greater diffusivity: those below 500 nm are typically internalized via endocytosis, whereas particles in the 0.5–2 μm range are preferentially taken up by RPE cells through phagocytosis [74]. Experimental data confirm these patterns—polystyrene nanoparticles of approximately 2 μm persist in the vitreous for up to one month, while 50–200 nm particles show vitreous half-lives of 8–10 days and successfully reach the retina [75].

Surface charge is another major determinant of nanoparticle fate, influencing electrostatic interactions with ocular tissues and carrier stability. The vitreous, ILM, and retinal cell membranes all possess a net negative charge. Consequently, highly cationic nanoparticles tend to interact strongly with these structures, which can impede vitreous diffusion and alter clearance dynamics [17]. Negatively or neutrally charged systems are more prone to rapid clearance due to charge-mediated destabilization and drainage. Koo et al. demonstrated that negatively charged human serum albumin (HSA) and HA-based carriers could cross the ILM and reach the RPE, whereas cationic polymers such as polyethylenimine (PEI) and glycol chitosan were retained within the vitreous [76]. Moderately cationic liposomes (~+20–30 mV) have been shown to diffuse through the vitreous and penetrate the retina, whereas highly cationic carriers (>+40 mV) become trapped at the ILM, underscoring the importance of balancing electrostatic interactions [77].

In systemic delivery via the choroidal vasculature, the fenestrated choriocapillaris provides potential access to the RPE, although the diaphragms spanning its 60–80 nm pores restrict nanoparticle passage to an effective size of ~6 nm. Transport across this barrier occurs by diffusion or through caveolae- or clathrin-mediated transcytosis [27]. Once extravasated, nanoparticles must traverse BM before contacting the RPE, where phagocytic uptake facilitates internalization. Very small dendrimers and gold nanoparticles can cross the choroid and reach the RPE, likely by passive diffusion, but typically lack tissue specificity, resulting in widespread retinal distribution [70,78].

The mechanical properties of nanocarriers also affect their intraocular dynamics. Deformable systems, such as nanogels, can adapt their shape to navigate extracellular matrices more efficiently than rigid particles, enhancing retinal penetration. Conversely, overly soft carriers may undergo rapid clearance, reducing residence time and bioavailability [76]. Once exposed to ocular fluids, nanoparticles rapidly adsorb proteins and biomolecules, forming a dynamic “protein corona” that modifies their size, surface charge, and biological identity [79]. This phenomenon influences uptake pathways and may even mask targeting ligands, altering biodistribution. Overall, both deformability and corona formation are critical determinants of nanocarrier performance, with softer structures such as nanogels and liposomes often showing superior barrier traversal compared to rigid counterparts [76].

### 6.2. Ligand–Receptor-Mediated Targeting

Surface functionalization of nanoparticles with specific ligands enables selective interaction with receptors expressed on RPE cells [17]. Representative ligand–receptor-mediated targeting strategies are summarized in Table 3.

Among these, HA has been extensively investigated for its affinity to CD44, a receptor expressed by the RPE and upregulated during inflammation. In preclinical models, HA-modified nanoparticles enhance endocytosis and improve delivery efficiency for both small molecules and nucleic acids. For example, HA-coated LNPs preferentially localized to the RPE–choroid complex following intravitreal injection in experimental autoimmune uveitis, persisting for several days; uptake efficiency correlated with HA molecular weight and surface density [80].

Similarly, HA-modified lipid-based nanoparticles enhanced gene delivery to RPE cells in vitro [69], while topically applied HA-coated gold nanoparticles reached posterior ocular tissues, including the RPE, highlighting their potential for noninvasive delivery [71].

Peptide ligands offer another versatile platform for RPE targeting. Cleavable peptide linkers responsive to lysosomal enzymes such as cathepsin D—abundantly expressed in RPE cells—enable selective intracellular drug release [81]. In addition, cell-penetrating peptides (CPPs), including trans-activator of transcription (TAT) and penetratin, can facilitate transport across the ILM and promote RPE uptake, although their lack of specificity and potential cytotoxicity remain concerns [82].

For systemic delivery, the RGD (arginine–glycine–aspartic acid) motif has been widely used to exploit integrin receptors highly expressed in the RPE and CNV regions under pathological conditions. In preclinical studies, RGD-functionalized PEI nanoparticles encapsulating antioxidants selectively accumulated in the RPE and CNV lesions, providing sustained release and therapeutic benefit in laser-induced CNV models [83]. Likewise, RGD-modified liposomes demonstrated efficient choroidal extravasation and enhanced RPE localization compared with unmodified carriers [68].

**Table 3:** Examples of ligand–receptor-mediated RPE-targeting strategies.

Ligand	Target Receptor/Mechanism	Nanoparticle Example	Key Outcome
Hyaluronic acid (HA)	CD44	HA-modified LNPs	Preferential localization in RPE–choroid complex after IVT injection; uptake influenced by HA molecular weight and surface density [69,80]
Hyaluronic acid (HA)	CD44	HA-coated gold nanoparticles (topical)	Reached retina after eye-drop administration; noninvasive delivery potential [71]
Cleavable peptide linker (cathepsin D–sensitive)	Cathepsin D (lysosomal enzyme, highly expressed in RPE)	Peptide-modified nanoparticles	Enabled selective intracellular drug release in RPE cells, reducing off-target effects [81]
RGD peptide (arginine–glycine–aspartic acid)	Integrins (αvβ3, others)	RGD-functionalized PEI nanoparticles	Selective accumulation in RPE and CNV regions; sustained release and therapeutic effect in CNV mouse model [83]

### 6.3. Pathology-Responsive Targeting

Pathology-responsive strategies harness disease-associated cues—such as oxidative stress, inflammation, and altered endo-lysosomal or redox states—to concentrate therapy at sites of retinal damage while sparing healthy tissue [84].

Magnetic nanoparticles (MNPs) naturally exhibit tropism for the RPE following intravitreal administration and can be further engineered for disease-responsive behavior. In rodent models, MNPs rapidly and persistently accumulate in the RPE. When functionalized with VEGF, they undergo transcytosis across RPE cells toward the choroid, an effect that can be modulated by external magnetic fields to enhance localization [85].

Stimuli-responsive provide an additional layer of control by releasing their payload in response to pathological pH or redox conditions. Dual pH-sensitive lipid/DNA nanoparticles containing cleavable hydrazone linkers improved endosomal escape and RPE-targeted gene expression in vivo [86], while ROS-responsive mesoporous silica platforms have been shown to attenuate oxidative injury and neovascularization in preclinical retinal models [84]. A recent study further demonstrated redox-sensitive liposomes incorporating diselenide-containing alkyl chains that enabled drug release under oxidative stress. These ~140 nm vesicles efficiently encapsulated N-acetylcysteine (NAC) and, in human stem cell-derived RPE cultures, restored ~90% metabolic activity under oxidative challenge compared to NAC alone or non-responsive formulations. Such “smart” redox-triggered nanocarriers highlight the potential of stimuli-responsive systems for targeted antioxidant therapy in retinal degeneration [87].

## 7. Therapeutic Payloads Delivered to and Through the RPE

The diversity of nanotechnology platforms for ocular delivery has enabled an equally broad range of therapeutic payloads to be directed toward the RPE. These include small molecules, biologics, and nucleic acids—each with distinct requirements for stability, release kinetics, and intracellular trafficking [17].

### 7.1. Small Molecules: Steroids and Antioxidants

Early applications of nanocarriers in RPE-related disease focused on the delivery of anti-inflammatory and antioxidant agents. Poly(amidoamine) dendrimers conjugated with corticosteroids such as fluocinolone acetonide or dexamethasone demonstrated selective accumulation in activated microglia and RPE cells in models of retinal degeneration. These formulations provided sustained release and prolonged intraocular residence, resulting in more effective attenuation of neuroinflammation and improved preservation of retinal structure compared with the free drugs [88]. Comparable outcomes have been shown with dendrimer–dexamethasone systems and hydrophobic small molecules encapsulated in HA-modified LNPs or niosomes, which enhance solubility and retention within RPE-rich tissues [89]. Such findings highlight the therapeutic value of nanocarrier conjugation, as it can convert short-acting anti-inflammatory agents into longer-lasting treatments capable of directly modulating pathogenic inflammation at the RPE level—addressing a key unmet clinical need [54].

Given the central role of oxidative stress in RPE dysfunction—particularly in AMD—antioxidant delivery has been extensively explored [8]. Carotenoids such as lutein and zeaxanthin, widely used in dry AMD, have been reformulated into nanocarrier systems to enhance bioavailability and tissue penetration. Penetratin-modified lutein nanoemulsion gels improved release kinetics, increased uptake in ARPE-19 cells, and reduced photooxidative damage by lowering ROS levels and apoptosis [90]. Osmogen-modified nanoemulsions and formulations containing isopropyl myristate and triacetin further improved lutein solubility and permeability, underscoring the versatility of nanoemulsions in optimizing carotenoid delivery [91].

Beyond conventional antioxidants, composite nanomaterials have been engineered to amplify redox defense. Pegylated melanin–cerium oxide nanoparticles, for instance, combine the lipofuscin-binding capacity of melanin with the regenerative redox cycling of cerium, mimicking superoxide dismutase and catalase activity. These multifunctional particles offer a synthetic alternative to the age-related decline of endogenous melanin in the RPE [92]. Similarly, cerium oxide nanoparticles (nanoceria) exhibit potent radical-scavenging activity: a single intravitreal injection reduced retinal ROS, downregulated VEGF, and suppressed neovascularization in *Vldlr^−^/^−^* mice [75]. Beyond inorganic nanozymes, polymeric formulations of natural compounds such as curcumin, resveratrol, and pentamethyl-6-chromanol have shown strong antioxidant and anti-inflammatory effects in human RPE cultures and experimental retinas [93]. Collectively, these formulations are relevant because they reinforce the RPE’s antioxidant defense through localized and sustained release. This represents a significant advance over current AREDS-based supplementation, which relies on oral administration and suffers from limited ocular bioavailability [94,95].

### 7.2. Biologics: Anti-Angiogenic Agents

The RPE is a central regulator of angiogenesis in the posterior eye. Under physiological conditions, it maintains choroidal vascular homeostasis through balanced secretion of pro- and anti-angiogenic factors. In pathological states such as neovascular AMD, this equilibrium is disrupted, leading to excessive VEGF release from RPE cells and consequent CNV. The resulting invasion of abnormal, leaky vessels through BM compromises retinal structure and the BRB, ultimately causing vision loss [1,8].

Anti-VEGF biologics—including bevacizumab, ranibizumab, and aflibercept—have transformed the management of neovascular AMD by suppressing angiogenesis. Nanotechnology-based delivery systems have been developed to extend the duration of anti-VEGF activity while maintaining efficacy at the RPE–choroid interface [8].

Bevacizumab-loaded PLGA nanoparticles, including albumin-modified variants, demonstrated prolonged intraocular pharmacokinetics in rabbit eyes, maintaining therapeutic concentrations after a single injection [51]. In laser-induced CNV models, these formulations significantly reduced neovascular lesion size and preserved retinal morphology and function, confirming their therapeutic potential [96]. In Vitro, PLGA/PEG-LA systems maintained bevacizumab bioactivity for over 90 days, suggesting that these carriers could support clinically relevant, long-term release profiles [97].

Although AMD remains the primary focus of anti-angiogenic nanomedicine research, similar mechanisms involving RPE-derived growth factors also contribute to PVR. In PVR, RPE cells undergo epithelial-to-mesenchymal transition and produce pro-fibrotic and angiogenic mediators that drive formation of contractile epiretinal membranes. Current management relies solely on surgery, as no pharmacological treatments are approved. Encouragingly, nanotechnology-based sustained-release implants containing anti-proliferative agents such as 5-fluorouridine have reduced retinal detachment rates in experimental models without evident toxicity. Advanced multi-drug PLGA implants combining 5-fluorouridine, triamcinolone, and tissue plasminogen activator demonstrated both safety and efficacy, preventing pathological scarring while preserving retinal integrity [98]. These findings are particularly relevant given that PVR is driven by multiple interacting cytokines and growth factors. The capacity to co-deliver agents with complementary mechanisms within a single nanocarrier or composite implant represents a key emerging direction—offering the potential to act on several pathogenic pathways simultaneously and thereby better prevent or attenuate PVR progression [99].

### 7.3. Nucleic Acids and Gene-Editing Cargo

In addition to small molecules and biologics, nucleic acids represent a major therapeutic payload class for RPE-targeted nanomedicine [11]. Unlike viral vectors, nanoparticle-based systems circumvent limitations such as immunogenicity, insertional mutagenesis, and restricted packaging capacity, while offering biodegradability, tunable release, and redosability [100]. The RPE is particularly amenable to genetic modulation—whether for gene silencing in neovascular diseases or gene replacement/editing in inherited disorders. Preclinical studies have shown that nanoparticles can transfect RPE cells both in vitro and in vivo, enabling sustained gene expression with minimal toxicity [101].

siRNA therapeutics have been widely applied to silence angiogenesis-related genes. Trimethyl chitosan–HA polyplexes carrying siRNA against VEGFR2 significantly reduced lesion size in laser-induced CNV rat models after intravitreal injection, demonstrating the feasibility of non-viral gene silencing in the outer retina. Lipid-based nanoparticles further enhanced siRNA stability and intracellular delivery [102]. PEGylated lipid nanoparticles loaded with siRNA targeting VEGFR1 effectively entered ARPE-19 cells, suppressed VEGFR1 expression, and reduced CNV area in vivo. Complementary studies using integrin-targeted liposomes confirmed enhanced VEGF knockdown in RPE, illustrating how receptor-mediated uptake amplifies siRNA efficacy [103].

mRNA delivery has gained momentum following the success of LNP-based vaccines, with similar principles now applied to ocular gene therapy. Ionizable lipid nanoparticles have enabled efficient delivery of mRNA to the retina. Chambers et al. demonstrated robust transgene expression after intravitreal administration of LNP–mRNA constructs in mouse retina and human RPE explants, with minimal inflammation [104]. 

Peptide-guided LNPs have since been engineered to redirect tropism toward RPE and photoreceptors, achieving high expression levels across multiple species, including non-human primates. These advances are particularly relevant for protein replacement in inherited retinal degenerations, where delivery of functional mRNA to the RPE could compensate for defective endogenous proteins [105].

Plasmid DNA (pDNA) nanocarriers offer another route to long-term gene expression. CK30PEG-compacted DNA nanoparticles achieved efficient transfer of therapeutic genes to RPE and photoreceptors, with expression persisting for weeks and without immune activation. In RPE65-deficient models of Leber congenital amaurosis, subretinal administration of compacted DNA nanoparticles restored visual cycle activity and improved retinal function [106]. Similarly, nanoparticles delivering plasmids encoding the *Rds* gene partially rescued photoreceptor structure and function in retinitis pigmentosa models [107]. Solid-lipid nanoparticles carrying plasmid DNA have also shown promise for RPE targeting, informing design principles for improved nuclear uptake and transcriptional efficiency [108].

CRISPR-based gene editing has recently been adapted for non-viral delivery using nanoparticle platforms. Lipid nanoparticles encapsulating Cas9 mRNA and single-guide RNAs (sgRNAs) achieved targeted editing within RPE cells, producing ~16% on-target modification. This finding is significant because it demonstrates, for the first time, that precise in situ genome correction in RPE cells can be accomplished without viral vectors, greatly improving both safety and reproducibility [55].

More advanced dynamically covalent LNPs have enabled delivery of CRISPR ribonucleoproteins (RNPs) to disrupt *VEGF-A* in RPE cells, significantly suppressing CNV in laser-induced models [97]. These studies collectively underscore the promise of nanoparticle-mediated gene editing as a next-generation therapeutic avenue for RPE-associated diseases [11].

## 8. Challenges and Translational Considerations

The translation of nanotechnology-based delivery systems for RPE-related diseases faces multifaceted obstacles that extend well beyond proof-of-concept success in preclinical models [18]. The eye provides an attractive target for localized therapy due to its relative immune privilege and accessibility, yet it also presents unique anatomical and physiological barriers. The vitreous humor, ILM, BM, and the complex architecture of retinal cell layers all limit nanoparticle mobility and uptake. Disease states such as inflammation, fibrosis, or neovascular remodeling further alter these barriers dynamically, introducing variability and unpredictability in nanoparticle performance [17].

Another critical limitation arises from interspecies differences in ocular anatomy and physiology. Many preclinical studies employ rodents, which differ markedly from human eyes in terms of vitreous volume, retinal thickness, and extracellular matrix composition. These disparities may lead to overestimation of nanoparticle penetration, retention, or clearance. Larger models (e.g., rabbits) are anatomically more comparable in some respects, though species-specific tear film and mucosal dynamics complicate extrapolation to humans [89]. Non-human primate models remain indispensable for evaluating safety, biodistribution, and efficacy under conditions more faithfully approximating human retinal pathology [109].

Safety and biocompatibility are central considerations in clinical translation [110]. Nanoparticles, by virtue of their small size, high surface area, and reactive surfaces, can engage in unanticipated interactions with ocular tissues, potentially inducing cytotoxicity, oxidative stress, or immune activation [111]. For instance, LNPs employed for mRNA delivery to the RPE have elicited transient inflammatory responses (e.g., microglial activation, cytokine release) [104]. Polymeric dendrimers can be chemically tuned to reduce toxicity, but dose-dependent adverse effects and long-term clearance remain incompletely characterized [48]. Inorganic nanomaterials such as cerium oxide nanoparticles, while offering durable antioxidant function, raise concerns of persistence and accumulation in ocular tissues [95]. Exosome- or extracellular vesicle–based carriers are generally more biocompatible, but challenges in reproducibility, isolation, scalability, and regulatory standardization still hinder their translation [36].

Manufacturing, scale-up, and regulatory compliance represent further bottlenecks. Multi-component nanosystems (e.g., peptide-functionalized LNPs, polymer–lipid hybrids) pose significant obstacles for reproducible, GMP-compliant production [112]. Ensuring batch-to-batch consistency, long-term stability, and sterility are nontrivial tasks. Moreover, the cost of development and manufacturing remains a formidable barrier, especially for personalized formulations or treatments targeting rare IRDs [113].

Finally, the choice of administration route is critical to translational feasibility [18]. Subretinal injection offers direct access to the RPE and is effective in gene therapy, but its invasiveness and surgical requirement limit its suitability for chronic treatments. Intravitreal injection, already a clinical standard for anti-VEGF therapy, is safer and broadly accepted, albeit challenging from the standpoint of nanoparticle penetration across vitreoretinal barriers. The suprachoroidal route has emerged more recently as a compromise—less invasive than subretinal, yet more directly adjacent to the choroid–RPE complex—though clinical evidence is still nascent [19].

## 9. Conclusions and Future Directions

The RPE plays a central role in major retinal diseases, yet current treatments are limited by short therapeutic duration and insufficient specificity. Nanotechnology-based delivery systems offer a means to improve drug stability, prolong intraocular residence, and enhance targeted delivery to the RPE. Preclinical studies have shown benefits such as reduced oxidative stress, suppression of neovascularization, and preservation of retinal structure, highlighting the therapeutic potential of these platforms.

However, important challenges remain. The anatomical barriers of the posterior segment, uncertainties regarding long-term biocompatibility, and limitations in scalable manufacturing continue to hinder clinical translation. To address these issues, future work should focus on the development of stimuli-responsive “smart” nanocarriers that release drugs in response to pathological cues such as oxidative stress or hypoxia, as well as on improving the predictiveness of preclinical models and standardized safety evaluations.

Continued interdisciplinary research, including advances in materials science, imaging, and computational modeling, will be key to refining these technologies and moving them closer to clinical application. With carefully guided development, nanomedicine has the potential to become a meaningful therapeutic option for RPE-associated retinal disorders.

## Figures and Tables

**Table 1:** Overview of ocular drug delivery routes and their key characteristics.

Route of Administration	Anatomical Access	Advantages	Limitations
Topical (eye drops)	Corneal and conjunctival surface; limited diffusion beyond anterior chamber	Noninvasive, easy to use, high patient compliance	Extremely low posterior penetration; <5% reaches intraocular tissues; unsuitable for RPE targeting
Periocular	Subconjunctival, sub-Tenon’s	Moderately invasive; bypasses corneal barrier	Limited scleral permeability; rapid clearance by choroidal circulation; poor RPE bioavailability
Suprachoroidal	Space between sclera and choroid	Minimally invasive; localized posterior delivery; reduced anterior exposure; suitable for sustained-release formulations	Requires specialized microneedle device; nonuniform drug distribution; rapid clearance through choriocapillaris
Intravitreal	Pars Plana → Vitreous cavity	Clinically established; outpatient procedure; repeatable; low complication rate; suitable for chronic therapy	Diffusion barriers (vitreous and ILM); vector dilution; repeated injections required; limited RPE penetration
Subretinal	Pars Plana Vitrectomy → Potential space between photoreceptors and RPE	Direct access to target cells; high bioavailability; reduced immune activation; long-lasting effect after single procedure	Surgically invasive (requires vitrectomy); risk of retinal tears or detachment; limited treatment area; requires expert surgeon
Systemic (oral or intravenous)	Vascular access → choriocapillaris	Noninvasive; allows repeated dosing	Poor ocular bioavailability; high systemic exposure and toxicity risk; limited permeability across BM

## Data Availability

No new data were created or analyzed in this study. Data sharing is not applicable to this article.
